# Erratum: “Fuentes-Guerra et al., Exogenously Driven Neural Reactivation of Spatially Matching Visual Working-Memory Contents”

**DOI:** 10.1523/ENEURO.0302-26.2026

**Published:** 2026-10-28

In the article “Exogenously Driven Neural Reactivation of Spatially Matching Visual Working-Memory Contents,” by Águeda Fuentes-Guerra, Elisa Martín-Arévalo, Freek van Ede, and Carlos González-García, which was published online on March 26, 2026, the funding information in the Acknowledgments appeared incorrectly. The authors apologize for the oversight, and the online version has been corrected.

The updated Acknowledgments should read:

*This work was supported by Grant PID2024-157672NB-I00 funded by MICIU/AEI/10.13039/501100011033 and by ERDF, EU, to E.M.-A.; Grant PID2020-116342GA-I00 funded by MICIU/AEI/10.13039/501100011033/FEDER, EU, to E.M.-A. and C.G.-G.; and Grant PID2023-149428NB-I00 funded by MICIU/AEI/10.13039/501100011033/FEDER, EU, to C.G.-G. C.G.-G. was also supported by Grant Ramón y Cajal RYC2021-033536-I funded by MCIN/AEI/10.13039/501100011033 and by the European Union Next Generation EU/PRTR. F.vE. was supported by an ERC Starting Grant from the European Research Council (MEMTICIPATION, 850636) and an NWO Vidi Grant from the Dutch Research Council (Grant Number 14721). A.F-G. was supported by Grant PRE2021-100351, funded by MCIN/AEI/10.13039/501100011033. Additionally, this publication was funded by the European Social Fund Plus (ESF+), Grant CEX2023-001312-M funded by MCIN/AEI/10.13039/501100011033, and Grant UCE-PP2023-11 from the University of Granada Excellence Unit Program. This work is part of the doctoral thesis of A.F-G., under the supervision of E.M-A. and C.G-G. During the preparation of this work, the authors used ChatGPT-4 (GPT-4-turbo), accessed via Microsoft Copilot, to assist with English language correction. The authors reviewed and edited the content as necessary and take full responsibility for the final version of the manuscript.*

